# Psychometric properties and criterion related validity of the Norwegian version of hospital survey on patient safety culture 2.0

**DOI:** 10.1186/s12913-024-11097-7

**Published:** 2024-05-18

**Authors:** Espen Olsen, Seth Ayisi Junior Addo, Susanne Sørensen Hernes, Marit Halonen Christiansen, Arvid Steinar Haugen, Ann-Chatrin Linqvist Leonardsen

**Affiliations:** 1 UiS Business School, Department of Innovation, Management and Marketing, University of Stavanger, Stavanger, Norway; 2 Hospital of Southern Norway, Flekkefjord, Norway; 3 Department of Clinical Sciences, University of Bergen, Bergen, Norway; 4 Department of Obstetrics and Gynecology, Stavanger University Hospital, Stavanger, Norway; 5 Faculty of Health Sciences Department of Nursing and Health Promotion Acute and Critical Illness, OsloMet - Oslo Metropolitan University, Oslo, Norway; 6 Department of Anaesthesia and Intensive Care, Haukeland University Hospital, Bergen, Norway; 7 Faculty of Health, Welfare and Organization, Østfold University College, Fredrikstad, Norway; 8 Department of anesthesia, Østfold Hospital Trust, Grålum, Norway

**Keywords:** Hospital survey on patient safety culture, Hospital, HSOPSC, Patient safety culture, Psychometric testing, Validation

## Abstract

**Background:**

Several studies have been conducted with the 1.0 version of the Hospital Survey on Patient Safety Culture (HSOPSC) in Norway and globally. The 2.0 version has not been translated and tested in Norwegian hospital settings. This study aims to 1) assess the psychometrics of the Norwegian version (N-HSOPSC 2.0), and 2) assess the criterion validity of the N-HSOPSC 2.0, adding two more outcomes, namely ‘pleasure of work’ and ‘turnover intention’.

**Methods:**

The HSOPSC 2.0 was translated using a sequential translation process. A convenience sample was used, inviting hospital staff from two hospitals (*N* = 1002) to participate in a cross-sectional questionnaire study. Data were analyzed using Mplus. The construct validity was tested with confirmatory factor analysis (CFA). Convergent validity was tested using Average Variance Explained (AVE), and internal consistency was tested with composite reliability (CR) and Cronbach’s alpha. Criterion related validity was tested with multiple linear regression.

**Results:**

The overall statistical results using the N-HSOPSC 2.0 indicate that the model fit based on CFA was acceptable. Five of the N-HSOPSC 2.0 dimensions had AVE scores below the 0.5 criterium. The CR criterium was meet on all dimensions except Teamwork (0.61). However, Teamwork was one of the most important and significant predictors of the outcomes. Regression models explained most variance related to patient safety rating (adjusted R^2^ = 0.38), followed by ‘turnover intention’ (adjusted R^2^ = 0.22), ‘pleasure at work’ (adjusted R^2^ = 0.14), and lastly, ‘number of reported events’ (adjusted R^2=^0.06).

**Conclusion:**

The N-HSOPSC 2.0 had acceptable construct validity and internal consistency when translated to Norwegian and tested among Norwegian staff in two hospitals. Hence, the instrument is appropriate for use in Norwegian hospital settings. The ten dimensions predicted most variance related to ‘overall patient safety’, and less related to ‘number of reported events’. In addition, the safety culture dimensions predicted ‘pleasure at work’ and ‘turnover intention’, which is not part of the original instrument.

**Supplementary Information:**

The online version contains supplementary material available at 10.1186/s12913-024-11097-7.

## Background

Patient harm due to unsafe care is a large and persistent global public health challenge and one of the leading causes of death and disability worldwide [1]. Improving safety in healthcare is central in governmental policies, though progress in delivering this has been modest [2]. Patient safety culture surveys have been the most frequently used approach to measure and monitor perception of safety culture [3]. Safety culture is defined as “the product of individual and group values, attitudes, perceptions, competencies and patterns of behavior that determine the commitment to, and the style and proficiency of, an organization’s health and safety management” [4]. Moreover, safety culture refers to the perceptions, beliefs, values, attitudes, and competencies within an organization pertaining to safety and prevention of harm [5]. The importance of measuring patient safety culture was underlined by the results in a 2023 scoping review, where 76 percent of the included studies observed associations between improved safety culture and reduction of adverse events [6].

To assess patient safety culture in hospitals the US Agency for Healthcare Research and Quality (AHRQ) launched the Hospital Survey on Patient Safety Culture (HSOPSC) version 1.0 in 2004 [7, 8]. Since then, HSOPSC 1.0 has become one of the most used tools to evaluate patient safety culture in hospitals, administered to approximately hundred countries and translated into 43 languages as of September 2022 [9]. HSOPSC 1.0 has generally been considered to be one of the most robust instrument measuring patient safety culture, and it has adequate psychometric properties [10]. In Norway, the first studies using N-HSOPSC 1.0 concluded that the psychometric properties of the instrument were satisfactory for use in Norwegian hospital settings [11–13]. A recent review of literature revealed 20 research articles using the N-HSOPSC 1.0 [14].

Studies of safety culture perceptions in hospitals require valid and psychometric sound instruments [12, 13, 15]. First, an accurate questionnaire structure should demonstrate a match between the theorized content structure and the actual content structure [16, 17]. Second, psychometric properties of instruments developed in one context is required to demonstrate appropriateness in other cultures and settings [16, 17]. Further, psychometric concepts need to demonstrate relationships with other related and valid criteria. For example, data on criterion validity can be compared with criteria data collected at the same time (concurrent validity) or with similar data from a later time point (predictive validity) [12, 16, 17]. Finally, researchers need to demonstrate a match between the content theorized to be related to the actual content in empirical data [15]. If these psychometric areas are not taken seriously, this may lead to many pitfalls both for researchers and practitioners [14]. Pitfalls might be imprecise diagnostics of the patient safety level and failure to evaluate effect of improvement initiative. Moreover, researchers can easily erroneously confirm or reject research hypothesis when applying invalid and inaccurate measurement tools.

Patient safety cannot be understood as an isolated phenomenon, but is influenced by general job characteristics and the well-being of the individual health care workers. Karsh et al. [18] found that positive staff perceptions of their work environment and low work pressure were significantly related to greater job satisfaction and work commitment. A direct association has also been reported between turnover and work strain, burnout and stress [19] Zarei et al. [20] showed a significant relationship between patient safety (safety climate) and unit type, job satisfaction, job interest, and stress in hospitals. This study also illustrated a strong relationship between lack of personal accomplishment, job satisfaction, job interest and stress. Also, there was a negative correlation between occupational burnout and safety climate, where a decrease in the latter was associated with an increase in the former. Hence, patient safety researchers should look at healthcare job characteristics in combination with patient safety culture.

Recently, the AHRQ revised the HSOPSC 1.0 to a 2.0 version, to improve the quality and relevance of the instrument. HSOPSC 2.0 is shorter, with 25 items removed or with changes made for response options and ten additional items added. HSOPSC 2.0 was validated during the revision process [21], but the psychometric qualities across cultures, countries and in different settings need further investigation. Consequently, the overall aim of this study was to investigate the psychometric properties of the HSOPSC 2.0 [21] (see supplement 1) in a Norwegian hospital setting. Specifically, the aims were to 1) assess the psychometrics of the Norwegian version (N-HSOPSC 2.0), and 2) assess the criterion validity of the N-HSOPSC 2.0, adding two more outcomes, namely’ pleasure of work’ and ‘turnover intention’.

## Methods

### Design

This study had cross‐sectional design, using a web-based survey solution called “Nettskjema” to distribute questionnaires in two Norwegian hospitals. The study adheres to The Strengthening the Reporting of Observational Studies in Epidemiology (STROBE)Statement guidelines for reporting observational studies [22].

### Translation of the HSOPSC 2.0

We conducted a «forward and backward» translation in-line with recommendations from Brislin [23]. First, the questionnaires were translated from English to Norwegian by a bilingual researcher. The Norwegian version was then translated back to English by another bilingual researcher. Thereafter, the semantic, idiopathic and conceptual equivalence between the two versions were compared by the research group, consisting of experienced researchers. The face value of the N-HSOPSC 2.0-version was considered to be adequate and the items lend themselves well to the corresponding latent concepts.

### Piloting

The N-HSOPSC 2.0 was pilot-tested with focus on content and face validity. Six randomly selected healthcare personnel were asked to assess whether the questionnaire was adequate, appropriate, and understandable regarding language, instructions, and scores. In addition, an expert group consisting of senior researchers (*n* = 4) and healthcare personnel (*n* = 6), with competence in patient safety culture was asked to assess the same.

### The questionnaire

The HSOSPS 2.0 (supplement 1) consists of 32 items using 5-point Likert-like scales of agreement (from 1 = strongly disagree to 5 = strongly agree) or frequency (from 1 = never to 5 = always), as well as an option for “does not apply/do now know”. The 32 items are distributed over ten dimensions. Additionally, 2-single item patient safety culture outcome measures, and 6-item background information measures are included. The patient safety culture single item outcome measures evaluate the overall ‘patient safety rating’ for the work area, and ‘reporting patient safety events’.

In addition to the N-HSOPSC 2.0, participants were asked to respond to three questions about their ‘pleasure at work’ (measure if staff enjoy their work, and are pleased with their work, scored from 1 = never, to 4 = always) [24], two questions about their ‘intention to quit’ (measure is staff are considering to quit their job, scored on a 5-point likert scale where 1 = strongly agree to 5 = strongly disagree) [25], as well as demographic variables (gender, age, professional background, primary work area, years of work experience).

### Participants and procedure

The data collection was conducted in two phases: the first phase (Nov-Dec 2021) at Hospital A and the second phase at Hospital B (Feb-March 2022)). We used a purposive sampling strategy: At Hospital A (two locations), all employees were invited to participate (*N* = 6648). This included clinical staff, administrators, managers, and technical staff. At Hospital B (three locations) all employees from the anesthesiology, intensive care and operation wards were invited to participate (*N* = 655).

The questionnaire was distributed by e-mail, including a link to a digital survey solution delivered by the University of Oslo, and gathered and stored on a safe research platform: TSD (services for sensitive data). This is a service with two-factor authentication, allowing data-sharing between the collaborating institutions without having to transfer data between them. The system allows for storage of indirectly identifying data, such as gender, age, profession and years of experience, as well as hospital. Reminders were sent out twice.

### Statistical analyses

Data were analyzed using Mplus. Normality was assessed for each item using skewness and kurtosis, where values between + 2 and -2 are deemed acceptable for normal distribution [26]. Missing value analysis was conducted using frequencies, to check the percentage of missing responses for each item. Correlations were assessed using Spearman’s correlation analysis, reported as Cronbach’s alpha.

Confirmatory factor analysis (CFA) was conducted to test the ten-dimension structure of the N-HSOPSC 2.0 using Mplus and Mplus Microsoft Excel Macros. The structure was then tested for fitness using Comparative Fit Index (CFI), Tucker-Lewis Index (TLI), Root Mean Square Error of Approximation (RMSEA) and Standardized Root Mean Square Residual (SRMR) [27]. Table 1 shows the fitness indices and acceptable thresholds.

**Table 1:** Fitness indices and acceptable thresholds [27]

**Fit indices**	**Acceptable thresholds**	
**CFI**	> 0.95, excellent	> 0.90, acceptable
**TLI**	> 0.95, excellent	> 0.90, acceptable
**RMSEA**	< 0 .06, excellent	0.06-.10, moderate
**SRMR**	< 0.05, excellent	< 0.08, moderate

*Abbreviations*: *CFI* Comparative Fit Index, *TLI* Tucker-Lewis Index, *RMSEA* Root Mean Square Error of Approximation, *SRMR*  Standardized Root Mean Square Residual

Reliability of the 10 predicting dimensions were also assessed using composite reliability (CR) values, where 0.7 or above is deemed acceptable for ascertaining internal consistency [25].

Convergent validity was assessed using the Average Variance Explained (AVE), where a value of at least 0.5 is deemed acceptable [28], indicating that at least 50 percent of the variance is explained by the items in a dimension. Criterion-related validity was tested using linear regression, adding ‘turnover intention’ and ‘pleasure at work’ to the two single item outcomes of the N-HSOPSC 2.0.

Internal consistency and reliability were assessed using Cronbach’s alpha, where values > 0.9 is assumed excellent, > 0.8 = good, > 0.7 = acceptable, > 0.6 = questionable, > 0.5 = poor and < 0.5 = unacceptable [29].

### Ethical considerations

The study was conducted in-line with principles for ethical research in the Declaration of Helsinki, and informed consent was obtained from all the participants [30]. Completed and submitted questionnaires were assumed as consent to participate. Data privacy protection was reviewed by the respective hospitals’ data privacy authority, and assessed by the Norwegian Center for Research Data (NSD, project number 322965).

## Results

### Sample

In total, 1002 participants responded to the questionnaire, representing a response rate of 12.6 percent. As seen in Table 2, 83.7% of the respondents worked in Hospital A and the remaining 16.3% in Hospital B. The majority of respondents (75.7%) were female, and 75.9 percent of respondents worked directly with patients.

**Table 2:** Sample characteristics

**Variables**	***n***	***%***
**Hospitals**		
Hospital A	839	83.7
Hospital B	163	16.3
**Gender**		
Female	758	75.7
**Direct contact with patients**		
Yes	760	75.9
No	167	16.7
It depends	74	7.4
**Age**		
18–24 years	22	2.2
25–34 years	176	17.7
35–44 years	237	23.8
45–54 years	318	32.0
55–64 years	215	21.6
65–74 years	26	2.6
**Working experience**		
0–10 years	461	48.0
11–20 years	252	26.2
21–30 years	173	18.0
31–40 years	68	7.1
41–50 years	7	.7
**Job position (%)**		
0–25	26	2.7
26–50	52	5.3
51–75	107	11.0
76–100	790	80.8
101–125	2	.2

The skewness and kurtosis were between + 2 and -2, indicating that the data were normally distributed. All items had less than two percent of missing values, hence no methods for calculating missing values were used.

### Correlations

Correlations and Cronbach’s alpha are displayed in Table 3.

**Table 3:** Correlations and Cronbach’s alpha values (diagonal)

	**1**	**2**	**3**	**4**	**5**	**6**	**7**	**8**	**9**	**10**	**11**	**12**	**13**	**14**	**15**	**16**
**1.Gender**	-															
**2. Age**	-.12**	-														
**3. Teamwork**	.02	.02	**(.58)**													
**4. Staffing and work pace**	-.03	.21**	.29**	**(.74)**												
**5. Organizational learning**	.05	.03	.43**	.45**	**(.71)**											
**6. Response to error**	.07	.03	.46**	.35**	.59**	**(.74)**										
**7. Supervisor, manager or leader support for patient safety**	.01	.03	.44**	.42**	.65**	.60**	**(.74)**									
**8. Communication about error**	.02	.03	.45**	.22**	.56**	.50**	.55**	**(.85)**								
**9. Communication openness**	.02	.03	.46**	.33**	.55**	.60**	.58**	.56**	**(.76)**							
**10. Reporting patient safety events**	.07*	.04	.11**	.23**	.29**	.23**	.20**	.12**	.23**	**(.86)**						
**11. Hospital management support for patient safety**	.01	.10**	.25**	.45**	.47**	.34**	.42**	.30**	.36**	.32**	**(.79)**					
**12. Handoffs and information exchange**	.04	.15**	.24**	.44**	.35**	.32**	.35**	.21**	.35**	.28**	.37**	**(.80)**				
**13. Number of Reported events**	.03	-.10**	-.08**	-.20**	-.11**	-.07*	-.14**	-.08*	-.09**	-.14**	-.16**	-.14**	-			
**14. Patient Safety Rating**	-.01	.10**	.39**	.49**	.49**	.42**	.47**	.42**	.39**	.19**	.35**	.37**	-.12**	-		
**15. Turnover intention**	-.02	-.15**	-.36**	-.31**	-.32**	-.34**	-.30**	-.27**	-.28**	-.08**	-.28**	-.17**	.11**	-.37**	**(.85)**	
**16. Pleasure at work**	.00	.10**	.30**	.24**	.27**	.26**	.28**	.19**	.22**	.09**	.16**	.13**	-.11**	.31**	-.51**	**(.77)**

*Abbreviations*; Gender coded male = 1, female = 2. Cronbach’s alphas boldened, diagonal

^*^p < .05

^**^p < .01

The following dimensions had the highest correlations; ‘teamwork’, ‘staffing and work pace’, ‘organizational learning-continuous improvement’, ‘response to error’, ‘supervisor support for patient safety’, ‘communication about error’ and ‘communication openness’. Only one dimension, ‘teamwork’ (0.58), had a Cronbach’s alpha below 0.7 (acceptable). Hence, most of the dimensions indicated adequate reliability. Higher levels of the 10 safety dimensions correlate positively with patient safety ratings.

### Confirmatory Factor Analysis (CFA)

Table 4 shows the results from the CFA. CFA (*N* = 1002) showed acceptable fitness values [CFI = 0.92, TLI = 0.90, RMSEA = 0.045, SRMR = 0.053] and factor loadings ranged from 0.51–0.89 (see Table 1). CR was above the 0.70 criterium on all dimensions except on ‘teamwork’ (0.61). AVE was above the 0.50 criterium except on ‘teamwork’ (0.35), ‘staffing and work pace’ (0.44), ‘organizational learning-continuous improvement’ (0.47), ‘response to error’ (0.47), and communication openness.

**Table 4:** Confirmatory factor analysis with standardized factor loadings

	Factor loadings	M	SD	AVE	CR	Missing values n (%)
**Teamwork**		4.20	.70	.35	.61	4 (0.4)
A1. In this unit, we work together as an effective team	.61	4.28	.84			6 (0.6)
A8. During busy times, staff in this unit help each other	.62	4.31	.84			11 (1.1)
A9. There is a problem with disrespectful behavior by those working in this unit	.51	3.99	1.11			10 (1.0)
**Staffing and work pace**		3.18	1.04	.44	.75	4 (0.4)
A2. In this unit, we have enough staff to handle the workload	.69	2.87	1.27			8 (0.8)
A3. Staff in this unit work longer hours than is best for patient care	.57	3.78	1.35			9 (0.9)
A5. This unit relies too much on temporary, float, or PRN staff	.54	3.04	1.55			10 (1.0)
A11. The work pace in this unit is so rushed that it negatively affects patient safety	.79	3.05	1.32			10 (1.0)
**Organizational learning-continuous improvement**		3.59	1.04	.47	.72	5 (0.5)
A4. This unit regularly reviews work processes to determine if changes are needed to improve patient safety	.67	3.57	1.26			9 (0.9)
A12. In this unit, changes to improve patient safety are evaluated to see how well they worked	.68	3.60	1.25			9 (0.9)
A14. This unit lets the same patient safety problems keep happening	.68	3.61	1.38			13 (1.3)
**Response to error**		3.96	.91	.47	.78	5 (0.5)
A6. In this unit, staff feel like their mistakes are held against them	.77	4.16	1.13			8 (0.8)
A7. When an event is reported in this unit, it feels like the person is being written up, not the problem	.77	3.94	1.21			8 (0.8)
A10. When staff make errors, this unit focuses on learning rather than blaming individuals	.60	4.01	1.06			13 (1.3)
A13. In this unit, there is a lack of support for staff involved in patient safety errors	.55	3.75	1.44			13 (1.3)
**Supervisor, manager, or clinical leader support for patient safety**		3.99	.89	.52	.76	5 (0.5)
B1. My supervisor, manager, or clinical leader seriously considers staff suggestions for improving patient safety	.77	4.13	1.07			16 (1.6)
B2. My supervisor, manager, or clinical leader wants us to work faster during busy times, even if it means taking shortcuts	.58	3.81	1.12			10 (1.0)
B3. My supervisor, manager, or clinical leader takes action to address patient safety concerns that are brought to their attention	.78	4.03	1.08			7 (0.7)
**Communication about error**		3.86	.98	.66	.86	5 (0.5)
C1. We are informed about errors that happen in this unit	.78	3.85	1.18			7 (0.7)
C2. When errors happen in this unit, we discuss ways to prevent them from happening again	.83	3.86	1.08			8 (0.8)
C3. In this unit, we are informed about changes that are made based on event reports	.82	3.88	1.10			9 (0.9)
**Communication openness**		4.06	.80	.45	.77	5 (0.5)
C4. In this unit, staff speak up if they see something that may negatively affect patient care	.63	4.16	.90			11 (1.1)
C5. When staff in this unit see someone with more authority doing something unsafe for patients, they speak up	.69	4.18	1.17			14 (1.4)
C6. When staff in this unit speak up, those with more authority are open to their patient safety concerns	.74	3.99	1.07			7 (0.7)
C7. In this unit, staff are afraid to ask questions when something does not seem right	.60	3.92	1.03			9 (0.9)
**Reporting patient safety events**		4.20	1.45	.77	.87	7 (0.7)
D1. When a mistake is caught and corrected before reaching the patient, how often is this reported?	.89	4.15	1.53			9 (0.9)
D2. When a mistake reaches the patient and could have harmed the patient, but did not, how often is this reported?	.85	4.24	1.55			10 (1.0)
**Hospital management support for patient safety**		2.99	1.21	.59	.81	5 (0.5)
F1. The actions of hospital management show that patient safety is a top priority	.82	3.16	1.34			10 (1.0)
F2. Hospital management provides adequate resources to improve patient safety	.84	2.74	1.45			12 (1.2)
F3. Hospital management seems interested in patient safety only after an adverse event happens	.61	3.04	1.47			12 (1.2)
**Handoffs and information exchange**		4.12	1.19	.62	.83	6 (0.6)
F4. When transferring patients from one unit to another, important information is often left out	.63	3.79	1.43			11 (1.1)
F5. During shift changes, important patient care information is often left out	.89	4.43	1.25			7 (0.7)
F6. During shift changes, there is adequate time to exchange all key patient care information	.81	4.15	1.51			9 (0.9)

*M* mean, *SD* standard deviation, *CR* composite reliability, *AVE* average variance explained

### Criterion validity

Independent dimensions of HSOPSC 2.0 were employed to predict four different criteria: 1) ‘number of reported events’, 2) ‘patient safety rating’, 3) ‘pleasure at work’, and 4) ‘turnover intentions’. The composite measures explained variance of all the outcome variables significantly thereby ascertaining criterion-related validity (Table 5). Regression models explained most variance related to ‘patient safety rating’ (adjusted R^2^ = 0.38), followed by ‘turnover intention’ (adjusted R^2^ = 0.22), ‘pleasure at work’ (adjusted R^2^ = 0.14), and lastly, number of reported events (adjusted R^2^ = 0.06).

**Table 5:** Regression models to assess the criterion-related validity

Predictors	Outcomes			
	Number of Reported events (Item D3)	Patient Safety Rating (Item E1)	Pleasure at work	Turnover intention
Teamwork	-.02	.13**	.23**	-.21**
Staffing and work pace	-.15**	.25**	.09*	-.14**
Organizational learning	.04	.10**	.06	-.06
Response to error	.04	.02	.06	-.11**
Supervisor support	-.05	.08*	.04	-.01
Communication about error	-.03	.15**	.01	-.03
Communication openness	.01	-.01	-.01	.02
Reporting patient safety events	-.08**	.03	-.00	.06*
Hospital management support	-.06	.03	-.01	-.11**
Handoffs and information exchange	-.03	.09**	-.02	.05
Adjusted R^2^	.06	.38	.14	.22

^**^p < .01

^*^p < .05

## Discussion

In this study we have investigated the psychometric properties of the N-HSOPSC 2.0. We found the face and content validity of the questionnaire satisfactory. Moreover, the overall statistical results indicate that the model fit based on CFA was acceptable. Five of the N-HSOPSC 2.0 dimensions had AVE scores below the 0.5 criterium, but we consider this to be the strictest criterium employed in the evaluations of the psychometric properties. The CR criterium was met on all dimensions except ‘teamwork’ (0.61). However, ‘teamwork’ was one of the most important and significant predictors of the outcomes. One the positive side, the CFA results supports the dimensional structure of N-HSOPSC 2.0, and the regression results indicate a satisfactory explanation of the outcomes. On the more critical side, particularly AVE scores reflect threshold below 0.5 on five dimensions, indicating items have certain levels of measurement error as well.

In our study, regression models explained most variance related to ‘patient safety rating’ (R^2^ = 0.38), followed by ‘turnover intention’ (R^2^ = 0. 22), ‘pleasure at work’ (R^2^ = 0.14), and lastly, number of reported events (R^2^ = 0.06). This supports the criterion validity of the independent dimensions of N-HSOSPC 2.0, also when adding ‘turnover intention’ and ‘pleasure at work’. These results confirm previous research on the original N-HSOPSC 1.0 [12, 13]. The current study also found that ‘number of reported events’ was negatively related to safety culture dimensions, which is also similar to the N-HSOPSC 1.0 findings [12, 13].

The current study did more psychometric assessments compared to the first Norwegian studies using HSOPSC 1.0 [11–13]. However, results from the current study still support that the overall reliability and validity of N-HSOPSC 2.0 when comparing the results with the first studies using N-HSOPSC 1.0 [11–13]. Also, based on theory and expectations, the dimensions predicted ‘pleasure at work’ and ‘overall safety rating’ positively, and ‘turnover intentions’ and ‘number of reported events’ negatively. The directions of the relations thereby support the overall criterion validity. Some of the dimensions do not predict the outcome variables significantly, nonetheless, each criterion related significantly to at least two dimensions on the HSOPSC 2.0. It is also worth noticing that ‘teamwork’ was generally one of the most important predictors even thought this dimension had the lowest convergent validity (AVE) in the previous findings [11–13], even if the strict AVE criterium was not satisfactory on the teamwork dimension and CR was also below 0.7. Since the explanatory power of teamwork was satisfactory, this illustrate that the AVE and CR criteria are maybe too strict.

The sample in the current study consisted of 1009 employees at two different hospital trusts in Norway and across different professions. The gender and ages are representative for Norwegian health care workers. In total 760 workers had direct patient contact, 167 had not, and 74 had patient contact sometimes. We think this mix is interesting, since a system perspective is key to establishing patient safety [31]. The other background variables (work experience, age, primary work area, and gender) indicate a satisfactory spread and mix of personnel in the sample, which is an advantage since then the sample to a large extend represent typical healthcare settings in Norway.

In the current study, N-HSOPSC 2.0 had higher levels of Cronbach’s alpha than in the first N-HSOPSC 1.0 studies [11, 13], but more in-line with results from a longitudinal Norwegian study using the N-HSOPSC 1.0 in 2009, 2010 and 2017 respectively [23]. Moreover, the estimates in the current study reveal a higher level of factor loading on the N-HSOPSC 2.0, ranging from 0.51 to 0.89. This is positive since CFA is a key method when assessing the construct validity [16, 17, 32].

AVE and CR were not estimated in the first Norwegian HSOPSC 1.0 studies [11, 13]. The results in this study indicate some issues regarding particularly AVE (convergent validity) since five of the concepts were below the recommended 0.50 threshold [32]. It is also worth noticing that all measures in the N-HSOPSC 2.0, except ‘teamwork’ (CR = 61), had CR values above 0.70, which is satisfactory. AVE is considered a strict and more conservative measure than CR. The validity of a construct may be adequate even though more than 50% of the variance is due to error [33]. Hence, some AVE values below 0.50 is not considered critical since the overall results are generally satisfactory.

The first estimate of the criterion related validity of the N-HSOPSC 2.0 using multiple regression indicated that two dimensions where significantly related to ‘number of reported events’, while six dimensions were significantly related to ‘patient safety rating’. The coefficients were negatively related with number of reported events, and positively related with patient safety rating, as expected. In the first Norwegian study in Norway on the N-HSOPSC 1.0 [13], five dimensions were significantly related to ‘number of reported events’, and seven dimensions were significantly related to ‘patient safety ratings’. The relations with ‘numbers of events reported’ were then both positive and negative, which is not optimal when assessing criterion validity. Hence, since all significant estimates are in the expected directions, the criterion validity of N-HSOPSC 2.0 has generally improved compared to the previous version.

In the current study we added ‘pleasure at work’ and ‘turnover intention’ to extend the assessment of criterion related validity. The first assessment indicated that ‘teamwork’ had a very substantial and positive influence on ‘pleasure at work’. Moreover, ‘staffing and work pace’ also had a positive influence on ‘pleasure at work’, but none of the other concepts were significant predictors. Hence, the teamwork dimension is key in driving ‘pleasure at work’, then followed by ‘staffing and working pace’. ‘Turnover intentions’ was significantly and negatively related to ‘teamwork’, ‘staffing and working pace’, ‘response to error’ and ‘hospital management support’. Hence, the results indicate these dimensions are key drivers in avoiding turnover intentions among staff in hospitals. A direct association has been reported between turnover and work strain, burnout and stress [19]. Zarei et al. [20] showed a significant relationship between patient safety (safety climate) and unit type, job satisfaction, job interest, and stress in hospitals. This study also illustrated a strong relationship between lack of personal accomplishment, job satisfaction, job interest and stress. Furthermore, a negative correlation between occupational burnout and safety climate was discovered, where a decrease in the latter is associated with an increase in the former [20]. Hence, patient safety researchers should look at health care job characteristics in combination with patient safety culture.

Assessment of psychometrics must consider other issues beyond statistical assessments such as theoretical consideration and face validity [16, 17]; we believe one of the strengths of the HSOPSC 1.0 is that the instrument was operationalized based on theoretical concepts. This has been a strength, as opposed to other instruments built on EFA and a random selection of items included in the development process. We believe this is also the case in relation to HSOPSC 2.0; the instrument is theoretically based, easy to understand, and most importantly, can function as a tool to improve patient safety in hospitals. Moreover, when assessing the items that belongs to the different latent constructs, item-dimension relationships indicate a high face validity.

Forthcoming studies should consider predicting other outcomes, such as for instance mortality, morbidity, length of stay and readmissions, with the use of N-HSOPSC 2.0.

### Limitations

This study is conducted in two Norwegian public hospital trusts, indicating some limitations about generalizability. The response rate within hospitals was low and therefore we could not benchmark subgroups. However, this was not part of the study objectives. The response rate may be hampered by the pandemic workload, and high workload in the hospitals. However, based on the diversity of the sample, we find the study results robust and adequate to explore the psychometric properties of N-HSOPSC 2.0. For the current study, we did not perform sample size calculations. With over 1000 respondents, we consider the sample size adequate to assess psychometric properties. Moreover, the low level of missing responses indicate N-HSOPSC 2.0 was relevant for the staff included in the study.

There are many alternative ways of exploring psychometric capabilities of instruments. For example, we did not investigate alternative factorial structures, e.g. including hierarchical factorial models or try to reduce the factorial structure which has been done with N-HSOPSC 1.0 short [34]. Lastly, we did not try to predict patient safety indicators over time using a longitudinal design and other objective patient safety indicators.

## Conclusion

The results from this study generally support the validity and reliability of the N-HSOPSC 2.0. Hence, we recommend that the N-HSOPSC 2.0 can be applied without any further adjustments. However, future studies should potentially develop structural models to strengthen the knowledge and relationship between the factors included in the N-HSOPSC 2.0/ HSOPSC 2.0. Both improvement initiatives and future research projects can consider including the ‘pleasure at work’ and ‘turnover intentions’ indicators, since N-HSOPSC 2.0 explain a substantial level of variance relating to these criteria. This result also indicates an overlap between general pleasure at work and patient safety culture which is important when trying to improve patient safety.

### Supplementary Information

Supplementary Material 1.

## Data Availability

Datasets generated and/or analyzed during the current study are not publicly available due to local ownership of data, but aggregated data are available from the corresponding author on reasonable request.
