# Supplementary material for: Psychometric properties and criterion related validity of the Norwegian version of hospital survey on patient safety culture 2.0
Source: BMC Health Serv Res. 2024 May 18;24:642. doi: 10.1186/s12913-024-11097-7 (PMC11102629; doi:10.1186/s12913-024-11097-7)
Supplement: Supplementary file 1 — Supplementary Material 1. [file 12913_2024_11097_MOESM1_ESM.pdf]

SOPS™ Hospital Survey

Version: 2.0
Language: English

Notes

- For more information on getting started, selecting a sample, determining data collection methods, establishing data collection procedures, conducting a web-based survey, preparing and analyzing data, and producing reports, please read the **Hospital Survey Version 2.0 User's Guide.**

- For the survey items grouped according to the safety culture composite measures they are intended to assess, please refer to the **Hospital Survey Version 2.0 Items and Composite Measures** document.

- To participate in the AHRQ Hospital Survey on Patient Safety Culture Database, you must have administered the survey in its entirety without significant modifications or deletions:
  - No changes to any of the survey item text and response options.
  - No reordering of survey items.
  - Questions added only at the end of the survey after Section F, before the background questions in Section G.

For assistance with this survey, please contact the SOPS Help Line at 1-888-324-9749 or SafetyCultureSurveys@westat.com.

Surveys on Patient Safety Culture™

Hospital Survey on Patient Safety (Version 2.0)

Instructions

This survey asks for your opinions about patient safety issues, medical error, and event reporting in your hospital and will take about 10-15 minutes to complete. If a question does not apply to you or your hospital or you don't know the answer, please select "Does Not Apply or Don't Know."

- ***"Patient safety"*** *is defined as the avoidance and prevention of patient injuries or adverse events resulting from the processes of healthcare delivery.*
- ***A "patient safety event"*** *is defined as any type of healthcare-related error, mistake, or incident, regardless of whether or not it results in patient harm.*

Your Staff Position

1. What is your position in this hospital?

Select ONE answer.

Nursing

- ☐1 Advanced Practice Nurse (NP, CRNA, CNS, CNM)
- ☐2 Licensed Vocational Nurse (LVN), Licensed Practical Nurse (LPN)
- ☐3 Patient Care Aide, Hospital Aide, Nursing Assistant
- ☐4 Registered Nurse (RN)

Medical

- ☐5 Physician Assistant
- ☐6 Resident, Intern
- ☐7 Physician, Attending, Hospitalist

Other Clinical Position

- ☐8 Dietitian
- ☐9 Pharmacist, Pharmacy Technician
- ☐10 Physical, Occupational, or Speech Therapist
- ☐11 Psychologist
- ☐12 Respiratory Therapist
- ☐13 Social Worker
- ☐14 Technologist, Technician (e.g., EKG, Lab, Radiology)

Supervisor, Manager, Clinical Leader, Senior Leader

- ☐15 Supervisor, Manager, Department Manager, Clinical Leader, Administrator, Director
- ☐16 Senior Leader, Executive, C-Suite

Support

- ☐17 Facilities
- ☐18 Food Services
- ☐19 Housekeeping, Environmental Services
- ☐20 Information Technology, Health Information Services, Clinical Informatics
- ☐21 Security
- ☐22 Transporter
- ☐23 Unit Clerk, Secretary, Receptionist, Office Staff

Other

- ☐24 Other, please specify:

Your Unit/Work Area

2. Think of your "unit" as the work area, department, or clinical area of the hospital where you spend *most* of your work time. What is your primary unit or work area in this hospital?

Select ONE answer.

Multiple Units, No specific unit
- ☐1 Many different hospital units, No specific unit

Medical/Surgical Units
- ☐2 Combined Medical/Surgical Unit
- ☐3 Medical Unit (Nonsurgical)
- ☐4 Surgical Unit

Patient Care Units
- ☐5 Cardiology
- ☐6 Emergency Department, Observation, Short Stay
- ☐7 Gastroenterology
- ☐8 ICU (All Adult Types)
- ☐9 Labor & Delivery, Obstetrics & Gynecology
- ☐10 Oncology, Hematology
- ☐11 Pediatrics (including NICU, PICU)
- ☐12 Psychiatry, Behavioral Health
- ☐13 Pulmonology
- ☐14 Rehabilitation, Physical Medicine
- ☐15 Telemetry

Surgical Services
- ☐16 Anesthesiology
- ☐17 Endoscopy, Colonoscopy
- ☐18 Pre Op, Operating Room/Suite, PACU/Post Op, Peri Op

Clinical Services
- ☐19 Pathology, Lab
- ☐20 Pharmacy
- ☐21 Radiology, Imaging
- ☐22 Respiratory Therapy
- ☐23 Social Services, Case Management, Discharge Planning

Administration/Management
- ☐24 Administration, Management
- ☐25 Financial Services, Billing
- ☐26 Human Resources, Training
- ☐27 Information Technology, Health Information Management, Clinical Informatics
- ☐28 Quality, Risk Management, Patient Safety

Support Services
- ☐29 Admitting/Registration
- ☐30 Food Services, Dietary
- ☐31 Housekeeping, Environmental Services, Facilities
- ☐32 Security Services
- ☐33 Transport

Other
- ☐34 Other, please specify:

SECTION A: Your Unit/Work Area

How much do you agree or disagree with the following statements about your unit/work area?

**Think about your unit/work area:**	**Strongly Disagree** ▼	**Disagree** ▼	**Neither Agree nor Disagree** ▼	**Agree** ▼	**Strongly Agree** ▼	**Does Not Apply or Don't Know** ▼
1. In this unit, we work together as an effective team	☐1	☐2	☐3	☐4	☐5	☐9
2. In this unit, we have enough staff to handle the workload	☐1	☐2	☐3	☐4	☐5	☐9
3. Staff in this unit work longer hours than is best for patient care	☐1	☐2	☐3	☐4	☐5	☐9
4. This unit regularly reviews work processes to determine if changes are needed to improve patient safety	☐1	☐2	☐3	☐4	☐5	☐9
5. This unit relies too much on temporary, float, or PRN staff	☐1	☐2	☐3	☐4	☐5	☐9
6. In this unit, staff feel like their mistakes are held against them	☐1	☐2	☐3	☐4	☐5	☐9
7. When an event is reported in this unit, it feels like the person is being written up, not the problem	☐1	☐2	☐3	☐4	☐5	☐9
8. During busy times, staff in this unit help each other	☐1	☐2	☐3	☐4	☐5	☐9
9. There is a problem with disrespectful behavior by those working in this unit	☐1	☐2	☐3	☐4	☐5	☐9
10. When staff make errors, this unit focuses on learning rather than blaming individuals	☐1	☐2	☐3	☐4	☐5	☐9
11. The work pace in this unit is so rushed that it negatively affects patient safety	☐1	☐2	☐3	☐4	☐5	☐9
12. In this unit, changes to improve patient safety are evaluated to see how well they worked	☐1	☐2	☐3	☐4	☐5	☐9
13. In this unit, there is a lack of support for staff involved in patient safety errors	☐1	☐2	☐3	☐4	☐5	☐9
14. This unit lets the same patient safety problems keep happening	☐1	☐2	☐3	☐4	☐5	☐9

SECTION B: Your Supervisor, Manager, or Clinical Leader

How much do you agree or disagree with the following statements about your immediate supervisor, manager, or clinical leader?

	Strongly Disagree ▼	Disagree ▼	Neither Agree nor Disagree ▼	Agree ▼	Strongly Agree ▼	Does Not Apply or Don't Know ▼
1. My supervisor, manager, or clinical leader seriously considers staff suggestions for improving patient safety	☐1	☐2	☐3	☐4	☐5	☐9
2. My supervisor, manager, or clinical leader wants us to work faster during busy times, even if it means taking shortcuts	☐1	☐2	☐3	☐4	☐5	☐9
3. My supervisor, manager, or clinical leader takes action to address patient safety concerns that are brought to their attention	☐1	☐2	☐3	☐4	☐5	☐9

SECTION C: Communication

How often do the following things happen in your unit/work area?

**Think about your unit/work area:**	Never ▼	Rarely ▼	Some-times ▼	Most of the Time ▼	Always ▼	Does Not Apply or Don't Know ▼
1. We are informed about errors that happen in this unit	☐1	☐2	☐3	☐4	☐5	☐9
2. When errors happen in this unit, we discuss ways to prevent them from happening again ..	☐1	☐2	☐3	☐4	☐5	☐9
3. In this unit, we are informed about changes that are made based on event reports	☐1	☐2	☐3	☐4	☐5	☐9
4. In this unit, staff speak up if they see something that may negatively affect patient care	☐1	☐2	☐3	☐4	☐5	☐9
5. When staff in this unit see someone with more authority doing something unsafe for patients, they speak up	☐1	☐2	☐3	☐4	☐5	☐9
6. When staff in this unit speak up, those with more authority are open to their patient safety concerns	☐1	☐2	☐3	☐4	☐5	☐9
7. In this unit, staff are afraid to ask questions when something does not seem right	☐1	☐2	☐3	☐4	☐5	☐9

SECTION D: Reporting Patient Safety Events

**Think about your unit/work area:**	**Never** ▼	**Rarely** ▼	**Some-times** ▼	**Most of the Time** ▼	**Always** ▼	**Does Not Apply or Don't Know** ▼
1. When a mistake is caught and corrected before reaching the patient, how often is this reported?	☐1	☐2	☐3	☐4	☐5	☐9
2. When a mistake reaches the patient and could have harmed the patient, but did not, how often is this reported?	☐1	☐2	☐3	☐4	☐5	☐9

3. In the past 12 months, how many patient safety events have you reported?

- ☐a. None
- ☐b. 1 to 2
- ☐c. 3 to 5
- ☐d. 6 to 10
- ☐e. 11 or more

SECTION E: Patient Safety Rating

1. How would you rate your unit/work area on patient safety?

Poor ▼	Fair ▼	Good ▼	Very Good ▼	Excellent ▼
☐1	☐2	☐3	☐4	☐5

SECTION F: Your Hospital

How much do you agree or disagree with the following statements about your hospital?

**Think about your hospital:**	**Strongly Disagree** ▼	**Disagree** ▼	**Neither Agree nor Disagree** ▼	**Agree** ▼	**Strongly Agree** ▼	**Does Not Apply or Don't Know** ▼
1. The actions of hospital management show that patient safety is a top priority	☐1	☐2	☐3	☐4	☐5	☐9
2. Hospital management provides adequate resources to improve patient safety	☐1	☐2	☐3	☐4	☐5	☐9
3. Hospital management seems interested in patient safety only after an adverse event happens	☐1	☐2	☐3	☐4	☐5	☐9
4. When transferring patients from one unit to another, important information is often left out	☐1	☐2	☐3	☐4	☐5	☐9
5. During shift changes, important patient care information is often left out	☐1	☐2	☐3	☐4	☐5	☐9
6. During shift changes, there is adequate time to exchange all key patient care information	☐1	☐2	☐3	☐4	☐5	☐9

SECTION G: Background Questions

1. How long have you worked in this <u>hospital</u>?

☐a. Less than 1 year
☐b. 1 to 5 years
☐c. 6 to 10 years
☐d. 11 or more years

2. In this hospital, how long have you worked in your current <u>unit/work area</u>?

☐a. Less than 1 year
☐b. 1 to 5 years
☐c. 6 to 10 years
☐d. 11 or more years

3. Typically, how many hours per week do you work in this <u>hospital</u>?

☐a. Less than 30 hours per week
☐b. 30 to 40 hours per week
☐c. More than 40 hours per week

4. In your staff position, do you typically have direct interaction or contact with patients?

☐a. YES, I typically have direct interaction or contact with patients
☐b. NO, I typically do NOT have direct interaction or contact with patients

SECTION H: Your Comments

Please feel free to provide any comments about how things are done or could be done in your hospital that might affect patient safety.

Thank you for completing this survey.
